# Supplementary material for: Short-Term Effects of Strengthening Exercises of the Lower Limb Rehabilitation Protocol on Pain, Stiffness, Physical Function, and Body Mass Index among Knee Osteoarthritis Participants Who Were Overweight or Obese: A Clinical Trial
Source: ScientificWorldJournal. 2021 Dec 22;2021:6672274. doi: 10.1155/2021/6672274 (PMC8716196; doi:10.1155/2021/6672274)
Supplement: Supplementary Materials — Additional File 1: trial protocol. Additional File 2: CONSORT 2010 checklist. Additional File 3: CONSORT 2010 flow diagram. [file 6672274.f1.zip › 6672274.f1/Additional file 1-Trial Protocol.pdf]

Clinical Trial Protocol
Iranian Registry of Clinical Trials

18 Apr 2021

Short term effects of strengthening exercises of lower limb rehabilitation protocol (LLRP) on pain, stiffness, physical function and body mass index (BMI) among knee osteoarthritis (OA) participants who are overweight or obese.

Protocol summary

Study aim

To determine the short term effects of strengthening exercises of lower limb rehabilitation protocol on pain, stiffness, physical function and body mass index among knee osteoarthritis participants who are overweight or obese

Design

The study will be single blinded randomized controlled design.Three research coordinators will be involved in data collection. The one coordinator will be involved in baseline data collection and two coordinators will be involved in the assessment of outcomes. Participants will be blinded of other groups intervention.

Settings and conduct

The study will be conducted at the urban area of Lahore Pakistan and measurements will be assessed at the Teaching Bay of Rehmatul-Lil-Alameen Postgraduate Institute

Participants/Inclusion and exclusion criteria

Inclusion Criteria: Overweight or obese knee osteoarthritis participants, age between 45 and 60 years, residing in the urban community of Lahore, second and third degree of knee changes according to Kellgren and Lawrence radio-graphic scale Exclusion Criteria: System lupus erythematosus, Flat feet, Spinal deformities, Participants having history of metabolic, hormonal, orthopaedic and cardiovascular diseases, Previous surgery of knee/s of any cause The patients who had any injections of knee/s in the last six months or Unable to walk independently

Intervention groups

Intervention 1: Intervention group 1 the strengthening exercises of the lower limbs and instructions of daily care. The training session will be of 30-40 minutes. It will consist of three times a week for 4 weeks at their houses. Intervention 2: Intervention group 2 the participants in this group will follow the instructions of daily care only as a usual care for duration of 4 weeks at their houses.

Main outcome variables

Primary: Knee pain, stiffness and physical function
Secondary: Patients, s satisfaction and exercise adherence

General information

Reason for update

Acronym

IRCT registration information

IRCT registration number: **IRCT20191221045846N2**
Registration date: **2020-06-28, 1399/04/08**
Registration timing: **prospective**

Last update: **2020-06-28, 1399/04/08**
Update count: **0**

Registration date

2020-06-28, 1399/04/08

Registrant information

Name

Muhammad Tariq Rafiq

Name of organization / entity

Punjab Employees Social Security Institution

Country

Pakistan

Phone

+92 42 99330101

Email address

drtariqrafiq149@yahoo.com

Recruitment status

Recruitment complete

Funding source

Expected recruitment start date
2020-07-10, 1399/04/20
Expected recruitment end date
2020-08-09, 1399/05/19
Actual recruitment start date
empty
Actual recruitment end date
empty
Trial completion date
empty

Scientific title
Short term effects of strengthening exercises of lower limb rehabilitation protocol (LLRP) on pain, stiffness, physical function and body mass index (BMI) among knee osteoarthritis (OA) participants who are overweight or obese.

Public title
Short term effects of exercises among knee osteoarthritis (OA) participants who are overweight or obese.
Purpose
Treatment
Inclusion/Exclusion criteria
Inclusion criteria:
Overweight or obese knee OA participants aged between 45 to 60 years. Familiar with WhatsApp application Participants will be chosen from the urban area community of Punjab Lahore, Pakistan. The sample included males and females having OA of one or both knees confirmed by medical specialist according to the Kellgren and Lawrence radiographic scale for the assessment of OA
Exclusion criteria:
Rheumatoid arthritis System lupus erythematosus Flat feet Spinal deformities Unable to walk independently History of metabolic, hormonal, orthopaedic or cardiovascular disease Previous surgery of knee/s Injections of knee/s for the last six months.
Age
From **45 years** old to **60 years** old
Gender
Both

Phase
2
Groups that have been masked

- Participant
- Outcome assessor
- Data analyser
- Data and Safety Monitoring Board

Sample size
Target sample size: **50**
Randomization (investigator's opinion)
Randomized
Randomization description
The researcher will allocate the selected knee osteoarthritis participants into two groups, namely, Rehabilitation Protocol Group (RPG) and Control Group (CG) by simple random technique using a computer-generated random number. The participants in the RPG will follow the strengthening exercise of LLRP and IDC for duration of 4-weeks. The participants in the CG will not involved in the rehabilitation protocols, but these participants will follow only the IDC for the duration of 4-weeks at their houses.
Blinding (investigator's opinion)
Single blinded
Blinding description
The principle investigator will not be blinded. The participants receiving the intervention will be blinded by simply not informing them of their treatment allocation. The coordinators collecting data will be independent individuals from trials and will be unaware of the group allocation. There will be different coordinators at the initial and final evaluation. Individuals performing the statistical analysis will be blinded by labelling the groups with non-identifying terms (such as X and Y).
Placebo
Not used
Assignment
Parallel
Other design features

Secondary Ids

empty

Ethics committees

1

Ethics committee
Name of ethics committee
Ethical Committee RAIC PESSI (Rehmatul-Lil-Alameen Postgraduate Institute of Cardiology, Punjab Empl
Street address
Multan Chongi Multan Road Lahore,
City
Lahore
Postal code
54000
Approval date
2020-05-20, 1399/02/31
Ethics committee reference number
RAIC PESSI/Estt/2020/36

Health conditions studied

1

Description of health condition studied
Osteoarthritis of knee
ICD-10 code
M17
ICD-10 code description
Osteoarthritis of knee

Primary outcomes

1

Description
knee pain

Timepoint
at baseline and after 4 weeks of intervention
Method of measurement
Western Ontario and McMaster Universities Osteoarthritis Index (WOMAC) score

2

Description
Stiffness
Timepoint
At baseline and after 4 weeks of intervention
Method of measurement
WOMAC score

3

Description
Physical function
Timepoint
At baseline and after 4 weeks of intervention
Method of measurement
WOMAC score

Secondary outcomes

1

Description
Body Mass Index
Timepoint
At baseline and after 4 weeks of intervention
Method of measurement
Calculated by the formula Weight (kg)/Height(meter square)

2

Description
Patients satisfaction
Timepoint
After 4 weeks of intervention
Method of measurement
Numeric rating scale ranging from 0 to 10

3

Description
Exercise adherence
Timepoint
After 4 weeks of intervention
Method of measurement
Numeric rating scale ranging from 0 to 10

Intervention groups

1

Description
The participants in this group (Rehabilitation Protocol Group) will perform the strengthening exercises of lower limb rehabilitation protocol (LLRP) and follow the instructions of daily care (IDC) for duration of four weeks at their houses according to the provided leaflet. Each training session of strengthening exercises will be performed three times a week for four weeks at their houses. These training sessions were the strengthening exercises of lower limbs in non-weight bearing sitting and lying positions. Each training session will consist of 45–60 minutes of training followed by 10 minutes warm up at the start and 10 minutes cool down at the end of training protocol.
Category
Treatment - Other

2

Description
The participants in this group (Control Group) will follow only the instructions of daily care (IDC) for duration of four weeks at their houses according to the provided leaflet.
Category
Treatment - Other

Recruitment centers

1

Recruitment center
Name of recruitment center
Teaching Bay of Rehmatul-Lil-Alameene Post Graduate Institute of Cardiology
Full name of responsible person
Dr Farid Ahmad Chaudhary
Street address
Teaching Bay of Rehmatul-Lil-Alameene Post Graduate Institute of Cardiology, Punjab Employees Social Security Institution, Multan Chongi, Multan Road Lahore
City
Lahore
Postal code
54000
Phone
+92 42 99330101
Fax
+92 42 99330098
Email
ms.raic@pessi.gop.pk

Sponsors / Funding sources

1

Sponsor
Name of organization / entity
Rehmatul-Lil-Alameene Post Graduate Institute of Cardiology
Full name of responsible person
Dr Farid Ahmad Chaudhary
Street address
Rehmatul-Lil-Alameene Post Graduate Institute of Cardiology, Punjab Employees Social Security

Institution, Multan Chongi, Multan Road Lahore
City
Lahore
Postal code
54000
Phone
+92 42 99330101
Fax
Email
ms.raic@pessi.gop.pk
Grant name
Grant code / Reference number
Is the source of funding the same sponsor organization/entity?
Yes
Title of funding source
Rehmatul-Lil-Alameene Post Graduate Institute of Cardiology
Proportion provided by this source
50
Public or private sector
Public
Domestic or foreign origin
Domestic
Category of foreign source of funding
empty
Country of origin
Type of organization providing the funding
Other

2

Sponsor
Name of organization / entity
University of Malaya
Full name of responsible person
Dr Eliza Hafiz
Street address
Centre for Sport and Exercise Sciences, University of Malaya, Kuala Lumpur, Malaysia
City
Kuala Lumpur
Postal code
42000
Phone
+60 3-7967 3327
Email
eliza@um.edu.my
Grant name
Grant code / Reference number
Is the source of funding the same sponsor organization/entity?
No
Title of funding source
N/A
Proportion provided by this source
50
Public or private sector
Public
Domestic or foreign origin
Foreign
Category of foreign source of funding
Sponsor: country of origin
Country of origin
PK
Type of organization providing the funding
Other

Person responsible for general inquiries

Contact
Name of organization / entity
Punjab Employees Social Security Institution
Full name of responsible person
Muhammad Tariq Rafiq
Position
Consultant Senior Physiotherapist
Latest degree
Medical doctor
Other areas of specialty/work
Physiotherapy
Street address
54000 B. Rehmatul-Lil-Alamine Post Graduate Institute of Cardiology, Punjab Employees Social Security Institution, Multan Chongi, Multan Road Lahore
City
Lahore
Province
Punjab
Postal code
54000
Phone
+92 42 99330101
Fax
Email
drtariqrafiq149@yahoo.com

Person responsible for scientific inquiries

Contact
Name of organization / entity
University of Malaya
Full name of responsible person
Mohamad Shariff A Hamid
Position
Associate Professor
Latest degree
Ph.D.
Other areas of specialty/work
Physical Medicine
Street address
Sports Medicine Department, University of Malaya Medical Centre, Kuala Lumpur, Malaysia
City
Kuala Lumpur
Province
Selangor
Postal code
59100
Phone
+60 3-7967 6669
Email
ayip@um.edu.my

Person responsible for updating data

Contact

Name of organization / entity
Punjab Employees Social Security Institution

Full name of responsible person
Muhammad Tariq Rafiq

Position
Consultant Senior Physiotherapist

Latest degree
Medical doctor

Other areas of specialty/work
Physiotherapy

Street address
54000 B. Rehmatul-Lil-Alamine Post Graduate Institute of Cardiology, Punjab Employees Social Security Institution, Multan Chongi, Multan Road Lahore

City
Lahore

Province
Punjab

Postal code
54000

Phone
+92 42 99330101

Fax

Email
drtariqrafiq149@yahoo.com

Sharing plan

Deidentified Individual Participant Data Set (IPD)
Undecided - It is not yet known if there will be a plan to make this available

Study Protocol
Undecided - It is not yet known if there will be a plan to make this available

Statistical Analysis Plan
Undecided - It is not yet known if there will be a plan to make this available

Informed Consent Form
Undecided - It is not yet known if there will be a plan to make this available

Clinical Study Report
Undecided - It is not yet known if there will be a plan to make this available

Analytic Code
Undecided - It is not yet known if there will be a plan to make this available

Data Dictionary
Undecided - It is not yet known if there will be a plan to make this available
