# Supplementary material for: Short-Term Effects of Strengthening Exercises of the Lower Limb Rehabilitation Protocol on Pain, Stiffness, Physical Function, and Body Mass Index among Knee Osteoarthritis Participants Who Were Overweight or Obese: A Clinical Trial
Source: ScientificWorldJournal. 2021 Dec 22;2021:6672274. doi: 10.1155/2021/6672274 (PMC8716196; doi:10.1155/2021/6672274)
Supplement: Supplementary Materials — Additional File 1: trial protocol. Additional File 2: CONSORT 2010 checklist. Additional File 3: CONSORT 2010 flow diagram. [file 6672274.f1.zip › 6672274.f1/Additional file 3-CONSORT-2010-Flow-Diagram.doc]

**CONSORT 2010 Flow Diagram**

**Allocation**

**Analysis**

**Follow-Up**

**Enrollment**

Assessed for eligibility (n= )

Excluded (n=	)

♦  Not meeting inclusion criteria (n= )

♦  Declined to participate (n= )

♦  Other reasons (n= )

Analysed (n= )
♦ Excluded from analysis (give reasons) (n= )

Lost to follow-up (give reasons) (n= )

Discontinued intervention (give reasons) (n= )

Allocated to intervention (n= )

♦ Received allocated intervention (n= )

♦ Did not receive allocated intervention (give reasons) (n= )

Lost to follow-up (give reasons) (n= )

Discontinued intervention (give reasons) (n= )

Allocated to intervention (n= )

♦ Received allocated intervention (n= )

♦ Did not receive allocated intervention (give reasons) (n= )

Analysed (n= )
♦ Excluded from analysis (give reasons) (n= )

Randomized (n= )
